# Vitamin D Supplementation Modestly Reduces Serum Iron Indices of Healthy Arab Adolescents

**DOI:** 10.3390/nu10121870

**Published:** 2018-12-02

**Authors:** Mohammad S. Masoud, Majed S. Alokail, Sobhy M. Yakout, Malak Nawaz K. Khattak, Marwan M. AlRehaili, Kaiser Wani, Nasser M. Al-Daghri

**Affiliations:** Chair for Biomarkers of Chronic Diseases, Biochemistry Department, College of Science, King Saud University, Riyadh 11451, Saudi Arabia; mohammad_masoud@hotmail.com (M.S.M.); msa85@yahoo.co.uk (M.S.A.); sobhy.yakout@gmail.com (S.M.Y.); malaknawaz@yahoo.com (M.N.K.K.); roony607@hotmail.com (M.M.A.); wani.kaiser@gmail.com (K.W.)

**Keywords:** serum iron, vitamin D, adolescents, Arab, vitamin D supplements

## Abstract

Vitamin D deficiency has been shown to affect iron status via decreased calcitriol production, translating to decreased erythropoiesis. The present study aimed to determine for the first time whether vitamin D supplementation can affect iron levels among Arab adolescents. A total of 125 out of the initial 200 Saudi adolescents with vitamin D deficiency (serum 25(OH)D < 50 nmol/L) were selected from the Vitamin D-School Project of King Saud University in Riyadh, Saudi Arabia. Cluster randomization was done in schools, and students received either vitamin D tablets (1000 IU/day) (*N* = 53, mean age 14.1 ± 1.0 years) or vitamin D-fortified milk (40IU/200mL) (*N* = 72, mean age 14.8 ± 1.4 years). Both groups received nutritional counseling. Anthropometrics, glucose, lipids, iron indices, and 25(OH)D were measured at baseline and after six months. Within group analysis showed that post-intervention, serum 25(OH)D significantly increased by as much as 50%, and a parallel decrease of −42% (*p*-values <0.001 and 0.002, respectively) was observed in serum iron in the tablet group. These changes were not observed in the control group. Between-group analysis showed a clinically significant increase in serum 25(OH)D (*p* = 0.001) and decrease in iron (*p* < 0.001) in the tablet group. The present findings suggest a possible inhibitory role of vitamin D supplementation in the iron indices of healthy adolescents whose 25(OH)D levels are sub-optimal but not severely deficient, implying that the causal relationship between both micronutrients may be dependent on the severity of deficiency, type of iron disorder, and other vascular conditions that are known to affect hematologic indices. Well-designed, randomized trials are needed to confirm the present findings.

## 1. Introduction

Through the last decade, vitamin D has gained considerable interest in health and biomedical research [1]. Globally, vitamin D deficiency is widespread and is considered a pandemic [2]. The Middle East and North African regions, including the Kingdom of Saudi Arabia (KSA), are not spared from this micronutrient deficiency, and in fact have among the highest rates of vitamin D deficiency in the world [3,4]. Among the most common risk factors for vitamin D deficiency in the Middle-East include female gender and their clothing style, multi-parity, sedentary lifestyle, urban living and socio-economic status for adults, and longer than average breastfeeding as well as low dietary vitamin D and calcium intake in children [5].

Vitamin D is involved in the proliferation and differentiation of bone marrow stem cells and may play a role in red cell production [6]. Vitamin D can also potentially affect circulating iron status by promoting erythropoiesis and by suppressing hepcidin expression [6]. Lower levels of pro-inflammatory cytokines and hepcidin increases iron bioavailability for erythropoiesis and hemoglobin synthesis by preventing iron sequestration in macrophages [7]. On the other hand, iron deficiency damages intestinal absorption of fat soluble vitamins, including vitamin D [8].

Similar to vitamin D deficiency, iron deficiency is also endemic and is, in fact, the most common micronutrient deficiency globally [9]. Adolescent girls are at high risk for iron deficiency because of diet and blood loss during menstruation [10]. Furthermore, according to the World Health Organization, the two main risk target groups for iron deficiency are pre-school children and young women [11,12]. It is also highly prevalent in infants, adolescents, and pregnant women and is believed to account for 75% of all types of anemia in the world, affecting 30% of population [13]. In the Middle East, the prevalence of anemia among women of child-bearing age is 47% in Egypt, 16% in Lebanon, 26.7% in the United Arab Emirates (UAE), and 40% in KSA [14].

Several cross-sectional studies confirm the association of vitamin D status and serum iron. In a large-scale study involving 2526 Korean children and adolescents, they observed that the occurrence of both vitamin D deficiency and anemia were significantly higher in females than males and concluded that the positive correlation between vitamin D and iron may be through the suppressive action of vitamin D in decreasing levels of hepcidin, an iron regulatory hormone [15]. Their results were consistent with the previous observations of Smith and colleagues among African Americans [7].

To date, observational and functional studies suggest a positive relationship between vitamin D status and serum iron, but interventional studies are lacking to prove causality. The recent meta-analysis of Azizi-Soleiman and colleagues indicated that iron supplementation trials conducted so far failed to improve vitamin D status [16], and limited data is available whether the reverse is true. Thus, the present interventional study aims to determine for the first time whether a vitamin D supplementation of six months duration can influence iron status among vitamin D deficient Saudi Arab adolescents.

## 2. Materials and Methods

### 2.1. Study Design and Participants

This was a 6-month follow up study involving 200 apparently healthy Saudi adolescents (100 boys and 100 girls) aged 13–17 years (overall mean age 14.1 ± 1.1 years; overall mean body mass index (BMI) 21.2 ± 0.8 kg/m^2^) with known vitamin D deficiency (serum 25(OH)D < 50 nmol/L) [17] at baseline and without medical conditions, such as asthma, hypertension, diabetes, liver, and renal diseases. The participants were taken from the Vitamin D School Project database of the Prince Mutaib Chair for Biomarkers of Osteoporosis (PMCO), King Saud University in Riyadh, Saudi Arabia [18,19].

In brief, the Vitamin D School Project is a collaborative project between King Saud University and the Ministry of Education in Riyadh, Saudi Arabia, ascertaining the beneficial effects of 1000 IU/day vitamin D supplementation and other vitamin D correction strategies, including vitamin D-fortified milk consumption and overall public health awareness in raising vitamin D levels. The project database includes information on more than 1000 students and teachers recruited from 34 different schools in the central region of Riyadh during winter-spring season (November–May 2014–2015), when sun exposure for optimum vitamin D_3_ production in Riyadh was observed to be shorter (10 A.M.–before 2 P.M.) than summer (9 A.M.–3 P.M.) [20]. Government-run school hours were from 6:30 A.M. until 1–2 P.M., Sunday-Thursday. Cluster randomization was done in the 34 schools. This type of randomization was done to prevent ‘contamination of allocation’, defined as participants in the control group being aware of the interventions given in the test group and adopting it themselves [21]. In the case of the present study, contamination of allocation can occur if both groups are in the same school, since the students in the control group can be influenced by peers/classmates to switch to the tablet group instead. Students from schools assigned to the milk (control) group were allocated to receive daily 200 mL of milk (per 100 mL contains 4.52 g carbohydrates, 3.22 g proteins, 3.0 g fats, 113 mg calcium, 40 IU of vitamin D, 102 IU of vitamin A, and 58 kcal) for 6 months. The milk provided was previously shown to have no effects in serum 25(OH)D levels [18]. Students from schools assigned to the tablet group received 1000 IU/day vitamin D supplementation (VitaD1000^®^, Synergy Pharma, Dubai, UAE) daily for 6 months. These interventions were monitored by their respective teachers and parents who were assigned to ensure that they were carried out daily in schooldays and weekends, respectively, for the entire duration of the study. Ethical approval was obtained from the Ethics Committee of the College of Science Research Center, King Saud University, Riyadh, Saudi Arabia (Ref No. 15/0502/IRB; Project No. E-15-1667), in accordance with the principles in the Declaration of Helsinki, as well as with the guidelines on good clinical practice. Prior to inclusion in the study, written informed consent was acquired from parents, as well as assent from the students.

**Figure 1:**
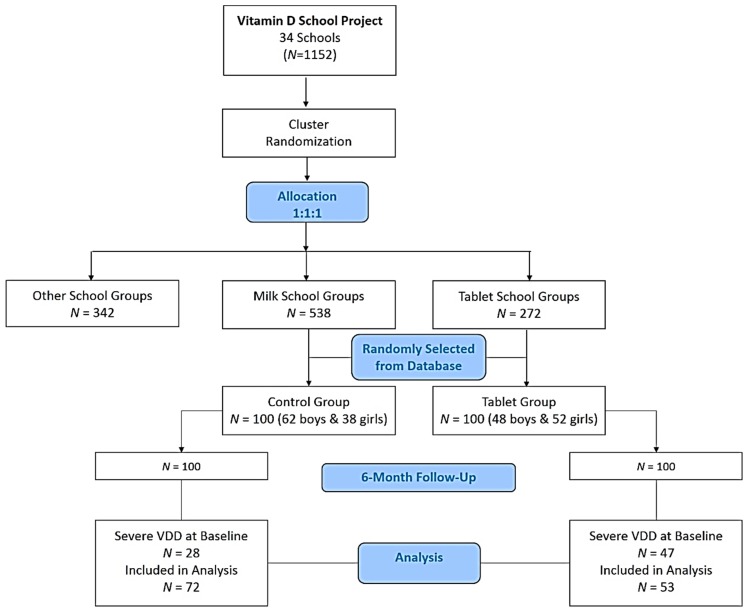
Study flowchart. VDD, vitamin D deficiency.

The cohort used in the present study were randomly selected from the school database of 2 groups (tablet group (*N* = 100); milk (control) group (*N* = 100)). Baseline characteristics of both groups are found in Supplementary Table S1. From the baseline assessment, significant differences were found in the prevalence of severe vitamin D deficiency (25(OH)D < 25 nmol/L) (tablet, 47%, versus control, 28%; *p* = 0.01) as well as baseline serum iron (*p* < 0.001), making the groups incomparable for prospective analysis. As extremely low levels of 25(OH)D can alter the overall metabolic profile, participants with severe vitamin D deficiency (25(OH)D <25nmol/L) were excluded in the analysis (*N* = 28 from the control, and *N* = 47 from the tablet group). The final overall sample size was *N* = 125. A flowchart has been provided in Figure 1.

### 2.2. Anthropometric and Biochemical Assessment

Information on anthropometrics (height, weight, body mass index, waist and hip circumference, waist-hip ratio, systolic and diastolic blood pressure) were extracted from the database to include values at baseline and after 6 months intervention. Anthropometrics were taken by an assigned research physician using a standard scale (Digital Pearson Scale, ADAM Equipment Inc., Oxford, CT, USA) for the assessment of height (cm) and weight (kg) measured in light clothing and without shoes. Waist and hip circumferences were measured using standard tape measure. Blood pressure (mmHg) was measured twice using a mercurial sphygmomanometer and the appropriate pediatric cuff. The average was noted. Body mass index (kg/m^2^) and waist-hip ratio (WHR) were calculated accordingly. Biochemical parameters for baseline and after 6 months, such as glucose, lipid profile (triglycerides, total cholesterol, LDL- and HDL-cholesterol), and calcium, were also retrieved from the database. Morning blood extraction was done twice (at baseline and after 6 months) for each participant after fasting for 8 h. Blood samples were centrifuged and delivered immediately in pre-labeled plain tubes, placed on ice, to King Saud University in Riyadh, Saudi Arabia, for storage and routine analysis using a biochemical analyzer (Konelab, Espoo, Finland).

### 2.3. Vitamin D and Iron Indices

Serum 25(OH) D was measured using COBAS e-411 automated analyzer (Roche Diagnostics, Indianapolis, IN, USA) in a DEQAS-certified laboratory (PMCO). Colorimetric ferrozine-based assay was used to measure iron and total iron-binding capacity in serum samples using a spectrophotometer. Transferrin saturation (%) was calculated as serum iron (µg/L)/total iron-binding capacity (TIBC) (µg/L) × 100.

### 2.4. Data Analysis

A G*power calculator was used for sample size determination. Using repeated measurement analysis, the observed effect size was 0.40 for a total sample size of 125, and the actual observed power was >0.85. Data were analyzed using SPSS (version 21) (IBM, Armonk, New York, USA). Continuous data were presented as mean ± standard deviation (SD) for variables following Gaussian variables, and non-Gaussian variables were presented in median (minimum-maximum). All continuous variables were checked for normality using Kolmogorov-Smirnov test, and non-normal variables were log-transformed. Categorical variables were presented in percentages (%) and Chi-square tests were performed. Independent *t*-test and paired *t*-test were used to check mean differences between group and time points respectively. Repeated measures analysis of co-variance (ANCOVA) was done to compare control and tablet groups. A *p*-value of <0.05 was considered statistically significant.

## 3. Results

Table 1 shows the general characteristics of the control and tablet groups after exclusion of participants with severe vitamin D deficiency at baseline. The control group had a higher systolic blood pressure than the tablet group, although this was borderline significant (*p* = 0.053). The rest of the baseline anthropometrics, biochemical indices, as well as serum 25(OH)D, calcium and iron indices were not significantly different between groups.

Table 2 shows the changes in the anthropometrics and routine biochemical indices over time. Within-group comparisons in the tablet group showed significant increases in waist circumference (*p* = 0.01) and waist-hip ratio (*p* < 0.001). There was also a significant decrease in glucose levels (*p* = 0.038) and triglycerides (*p* = 0.015) over time, parallel to the improvement, although borderline significant, in high density lipoprotein (HDL)-cholesterol levels (*p* = 0.06). Within-group comparison in the control group showed an overall increase in anthropometrics, including weight (*p* = 0.006), BMI (*p* = 0.09), hips (*p* = 0.049), and waist-hip ratio (*p* = 0.011). There was also a significant decrease in HDL-cholesterol levels over time in the control group (*p* = 0.008). Between-group comparisons showed a clinically significant difference in systolic blood pressure (*p* = 0.006), glucose (*p* = 0.029), triglycerides (*p* = 0.059), and HDL-cholesterol (*p* = 0.005) in favor of the tablet group. The rest of the between-group comparisons were not significant (Table 2).

Table 3 shows the effects of vitamin D supplementation in vitamin D status and iron indices over time. Within-group comparison showed a significant increase in 25(OH)D levels in the tablet group by as much as 50% (*p* < 0.001). Also in the tablet group, a significant decrease in iron levels was observed (−42%; *p* = 0.002), as well as in transferrin saturation (*p* = 0.01), parallel to the significant increase in TIBC (8.5%; *p* = 0.01). No significant changes were found in the control group. Between-group comparisons revealed a clinically significant increase in 25(OH)D levels in favor of the tablet group (*p* = 0.001) as well as a clinically significant reduction in iron (*p* < 0.001) and transferrin saturation levels (*p* = 0.005) (Table 3).

## 4. Discussion

The present interventional study evaluated the changes in circulating serum iron levels and other iron indices in a cohort of Saudi adolescents with suboptimal vitamin D levels before and after six months of daily 1000 IU vitamin D supplementation, as compared to controls. The present study is the first among Arab adolescents in prospectively determining the association between vitamin D and iron status. Among the highlights of the study are the clinically significant decrease in serum iron and transferrin saturation levels post-intervention in the tablet group, parallel to the significant improvements observed in blood pressure, glucose, and selected lipids, also in favor of the tablet group.

The counterintuitive effect of vitamin D supplementation in serum iron levels observed in the present study is in alignment with the observations of Doudin and colleagues conducted among >5000 healthy German adolescents of similar age groups, in that vitamin D levels seems to have an inhibitory role in hematological parameters, including hemoglobin where the bulk of iron is stored [22]. On the other hand, the clinical trial done by Madar and colleagues on healthy adults found that while four months of vitamin D3 (25 µg or 1000 IU) supplementation did not significantly affect any of the iron markers, the observed percentage changes post-intervention in hemoglobin (−0.6%), ferritin (−35%), and iron (−5.9%) were all trending downwards, similar to the present study [23]. Other clinical trials also found no significant changes in iron indices among healthy adults despite mega-doses of vitamin D3 [24,25]. Jastrzebska and colleagues have even taken into consideration the influence of physical activity and intermittent training since it can potentially affect vitamin D and iron metabolism, yet no significant differences were still found in the hematological parameters (Hb, Hct, and ferritin) of athletes given 5000 IU of vitamin D daily for eight weeks over those who did not receive supplementation [26]. All these previous studies, including the present one, suggest that vitamin D correction is unlikely to improve, if not reduce, iron indices, at least in apparently healthy populations.

Given the negative results of previous clinical trials among healthy subjects and the inhibitory effects found in the present study, it appears that the role of vitamin D supplementation in improving iron stores may be limited to those with certain metabolic conditions, such as those with poor vascular and renal function [27,28]. Suboptimal levels of both vitamin D and iron are biomarkers of ill health, and the hypothetical association appears to be reciprocal, as clinical observations demonstrated the role of 1, 25(OH)D in erythropoiesis, and the participation of iron is essential in the second activation of vitamin D in order to be functional [29,30]. The effect of vitamin D in reducing iron levels as observed in the present study, at least in participants who are apparently healthy and with no known vascular diseases, seem to support the mechanistic role of vitamin D as a chemopreventive agent in inhibiting erythropoiesis and angiogenesis, that in turn, suppresses proliferation of certain types of cells, including cancer cells [31].

Other findings include a general improvement in glucose and select lipids in favor of the tablet group. The mean serum vitamin D also significantly increased and almost reached a sufficient level (vitamin D ≥ 50 nmol/L) at follow-up. These changes were in alignment with previous vitamin D studies done in the KSA adolescent population [18,19]. Changes in selected anthropometric measures in both groups can be partially explained by dietary intake and physical activity which, unfortunately, were not taken into account in the present study.

The present findings should be interpreted taking into consideration its limitations. The present study is not a randomized controlled trial, and as such, several biases are evident due to non-randomization of participants and the lack of a better placebo group. Differences in baseline characteristics between the tablet and the control groups were minimized by removing all participants with baseline severe vitamin D deficiency. This finally gave a more comparable baseline metabolic profile as the 25(OH)D range was narrowed down (25–50 nmol/L).

Another major limitation is that the control group was given vitamin D-fortified milk, and dairy products have been observed to affect iron absorption due to their calcium content. The effects of dairy products in iron absorption, however, is still debatable since several intervention studies showed no significant change in iron indices from dairy product consumption [32,33]. Current recommendations, however, in milk consumption without affecting vitamin D and iron stores in children are 2 cups (500 mL) per day [34]. The control group in the present study were consuming only 200 mL/day. Serum calcium were also unaffected in both groups. More importantly, the vitamin D and calcium content in the milk products sold in Saudi Arabia are much lower than what the labels claim to be [35].

Other factors, such as dietary intake as a whole, as well as vitamin D intake and sunlight exposure, were also not taken into consideration and can significantly influence vitamin D status independent of the intervention assigned. However, epidemiologic observations done in Arab adolescents of Riyadh show that the majority have darker complexion, are fully clothed during outside activities (especially females), and prefer sunlight exposure before 10 A.M. [36]. These factors significantly reduce any clinically meaningful vitamin D conversion through sunlight exposure in this age group, especially in the present study, which was conducted during winter time when optimum sun light is not only reduced, but the best time to get sun exposure also falls well within their school hours. Other important parameters could not be analyzed, such as hepcidin and ferritin. Hepcidin, in particular, as a master regulator for iron absorption, has been shown to distinguish iron deficiency anemia and anemia of inflammation [37,38], with the latter type of anemia possibly benefiting more from vitamin D correction than the former [39,40].

Despite these limitations, the study remained sufficiently powered and adds value, as it documents the modest but significant effects of vitamin D supplementation in terms of influencing iron status in a relatively understudied ethnic population and age group. To the best of our knowledge, this is also the longest intervention trial done to determine changes in serum iron levels secondary to vitamin D supplementation. As the majority of the limited interventional studies also yielded negative results, given the clear association between vitamin D deficiency and risk of anemia [41,42], identifying which type of anemia will benefit from vitamin D supplementation might give more conclusive evidence.

## 5. Conclusions

In conclusion, a six-month vitamin D supplementation of 1000 IU/day significantly improved vitamin D status and consequently decreased serum iron levels among Saudi adolescents whose 25(OH)D levels are sub-optimal but not severely deficient. The study adds to the growing literature of the inhibitory and limited effects of vitamin D correction in the iron status of healthy individuals. The identification of the cause of iron deficiency is essential as to which demographics will benefit the most from vitamin D supplementation in terms of improving iron status. Well-designed and adequately powered randomized controlled trials including other iron indices, such as hepcidin, are encouraged to confirm present results. 

## Figures and Tables

**Table 1:** Baseline characteristics of intervention and control groups.

Parameter	Tablet	Control	*p*-Value
*N*	53	72	
Males (%)	30 (56.6)	40 (55.6)	0.68
**Anthropometrics**			
Age (years)	14.1 ± 1.0	14.8 ± 1.4	0.09
BMI (kg/m^2^)	22.8 ± 5.8	22.9 ± 6.2	0.96
Waist circumference (cm)	78.2 ± 15.8	80.1 ± 16.8	0.60
Hip Circumference (cm)	92.3 ± 13.8	94.5 ± 15.9	0.50
Waist-Hip Ratio	0.80 ± 0.1	0.80 ± 0.1	0.98
Systolic Blood Pressure (mmHg)	116.2 ± 12.9	122.5 ± 16.6	0.05
Diastolic Blood Pressure (mmHg)	70.6 ± 11.6	71.1 ± 13.6	0.88
**Routine Biochemical Indices**			
Glucose (mmol/L)	5.2 ± 0.6	5.4 ± 0.7	0.17
Triglycerides (mmol/L)	1.2 ± 0.6	1.3 ± 0.6	0.43
Total Cholesterol (mmol/L)	4.7 ± 0.8	4.5 ± 1.0	0.22
LDL-Cholesterol (mmol/L)	2.9 ± 0.7	2.5 ± 0.8	0.06
HDL-Cholesterol (mmol/L)	1.1 ± 0.3	1.3 ± 0.3	0.10
Calcium (mmol/L)	2.0 ± 0.1	1.9 ± 0.3	0.11
**Vitamin D and Iron Indices**			
25(OH)D (nmol/L)	34.6 ± 6.4	37.2 ± 7.5	0.09
Iron (µmol/L) #	18.2 (3–41)	21.5 (8–39)	0.09
Transferrin Iron-Binding Capacity (µmol/L) #	83.4 (19–102)	83.6 (28–99)	0.35
Transferrin Saturation (%) #	23.9 (3–71)	26.3 (2–70)	0.91

Note: # presented as median (interquartile range); *p*-value significant at <0.05.

**Table 2:** Changes in anthropometric and clinical parameters at baseline and follow-up.

Parameter	Tablet			Control			Tablet Effects
*N*	53			72			
	Baseline	Follow-Up	*p*-Value	Baseline	Follow-Up	*p*-Value	*p*-Value
**Anthropometrics**							
Weight (kg)	55.9 ± 17.3	56.3 ± 18.6	0.65	62.1 ± 19.3	65.0 ± 22.9	0.006	0.07
BMI (kg/m^2^)	22.8 ± 5.8	22.9 ± 6.2	0.59	22.9 ± 6.2	23.9 ± 7.5	0.09	0.69
Waist circumference (cm)	78.2 ± 15.8	82.2 ± 17.1	0.01	80.1 ± 16.8	81.4 ± 18.0	0.29	0.87
Hip circumference (cm)	92.3 ± 13.8	91.0 ± 13.4	0.17	94.5 ± 15.9	92.4 ± 14.4	0.049	0.56
Waist-Hip Ratio	0.8 ± 0.1	0.9 ± 0.1	<0.001	0.8 ± 0.1	0.9 ± 0.1	0.011	0.67
SBP (mmHg)	116.2 ± 12.9	113.9 ± 12.3	0.27	122.5 ± 16.6	121.4 ± 13.0	0.61	0.006
DBP (mmHg)	70.6 ± 11.6	69.8 ± 12.6	0.68	71.1 ± 13.6	69.9 ± 15.5	0.6	0.92
**Routine Biochemical Indices**							
Glucose (mmol/L)	5.2 ± 0.6	5.0 ± 0.5	0.038	5.4 ± 0.7	5.2 ± 0.7	0.15	0.029
Triglycerides (mmol/L) #	1.0 (0.3–3.1)	0.9 (0.3–2.3)	0.015	1.2 (0.3–3.1)	1.3 (0.4–3.0)	0.45	0.059
Total Cholesterol (mmol/L)	4.7 ± 0.8	4.7 ± 0.8	0.84	4.5 ± 1.0	4.6 ± 1.0	0.22	0.33
LDL-Cholesterol (mmol/L)	2.9 ± 0.7	2.8 ± 0.7	0.62	2.4 ± 0.8	2.5 ± 0.7	0.53	0.74
HDL-Cholesterol (mmol/L)	1.1 ± 0.3	1.3 ± 0.3	0.06	1.3 ± 0.3	1.1 ± 0.2	0.008	0.005
Calcium (mmol/L)	2.0 ± 0.2	1.9 ± 0.2	0.07	1.9 ± 0.5	1.8 ± 0.5	0.44	0.062

Note: # presented as median (min-max); significant at *p* < 0.05.

**Table 3:** Changes in vitamin D and iron indices at baseline and follow-up.

Parameters	Tablet (*N* = 53)			Control (*N* = 72)			Intervention Effects
	Baseline	Follow-Up	*p*-Value	Baseline	Follow-Up	*p*-Value	
25(OH)D (nmol/L)	34.6 ± 6.4	51.9 ± 13.0	<0.001	37.2 ± 7.5	37.9 ± 10.6	0.69	0.001
Iron (µmol/L) #	18.2 (2.1–40.9)	11.5 (1.3–49.5)	0.002	21.5 (8.1–39.5)	21.7 (8.7–38.0)	0.86	<0.001
TIBC (µmol/L) #	83.4 (18.7–102.8)	90.5 (78.9–102.5)	0.01	83.6 (28.0–99.5)	84.9 (52.7–99.5)	0.90	0.42
Transferrin Saturation (%) #	23.9 (2.1–70.8)	12.3 (1.4–48.7)	0.001	26.3 (1.2–70.7)	25.1 (10.3–80.3)	0.70	0.005

Note: # presented as median (min-max); significant at *p* < 0.05.

## Supplementary Materials

The following are available online at http://www.mdpi.com/2072-6643/10/12/1870/s1, Table S1: Baseline characteristics of intervention and control groups before exclusion of participants with severe vitamin D deficiency (25(OH)D <25nmol/L).
