# Supplementary material for: Vitamin D Supplementation Modestly Reduces Serum Iron Indices of Healthy Arab Adolescents
Source: Nutrients. 2018 Dec 2;10(12):1870. doi: 10.3390/nu10121870 (PMC6315440; doi:10.3390/nu10121870)
Supplement: Supplementary file 1 [file nutrients-10-01870-s001.pdf]

Supplemental Table 1. Baseline Characteristics of Intervention and Control Groups Before Exclusion of Participants with Severe Vitamin D Deficiency (25(OH)D <25nmol/L)

**Parameter**	**Tablet**	**Control**	***p*-Value**
N	100	100	
Males **(%)**	42 (42.0)	62 (62.0)	0.038
Severe 25(OH)D Deficiency (<25nmol/l)	47 (47.0)	28 (28.0)	0.011
**Anthropometrics**			
Age (years)	14.4 ± 1.0	14.8 ± 1.4	0.065
BMI (kg/m^2)	22.1 ± 5.8	23.5 ± 6.6	0.24
Waist circumference (cm)	73.7 ± 13.1	79.4 ± 16.6	0.053
Hip Circumference (cm)	91.7 ± 11.9	93.8 ± 15.2	0.44
Waist-Hip Ratio	0.80 ± 0.1	0.85 ± 0.1	0.034
Systolic Blood Pressure (mmHg)	115.9 ± 13.9	123.6 ± 15.9	0.005
Diastolic Blood Pressure (mmHg)	68.7 ± 10.8	72.1 ± 14.8	0.161
**Routine Biochemical Indices**			
Glucose (mmol/l)	5.1 ± 0.6	5.4 ± 0.6	0.02
Triglycerides (mmol/l)#	0.9 (0.3-3.1)	1.2 (0.4-3.1)	0.15
Total Cholesterol (mmol/l)	4.7 ± 0.8	4.5 ± 0.9	0.41
LDL-Cholesterol (mmol/l)	3.1 ± 0.7	2.5 ± 0.7	0.001
HDL-Cholesterol (mmol/l)	1.2 ± 0.3	1.3 ± 0.3	0.30
Calcium (mmol/l)	2.2 ± 0.4	1.7 ± 0.4	<0.001
**Vitamin D and Iron Indices**			
25(OH)D (nmol/l)	26.7 ± 7.9	29.2 ± 7.0	0.10
Iron (µmol/L) #	9.7 (2.0-24.0)	19.9 (11.3- 27.8)	<0.001
Transferrin Iron-Binding Capacity (µmol/L) #	62.0 (18.7-100.8)	81.6 (20.2-99.5)	0.21
Transferrin Saturation (%)#	23.3 (2.0-79.8)	25.9 (2.1-154.1)	0.51

Note: # presented as median (min-max); p-value significant at <0.05.
